# Morphological analysis of the filamentous fungus *Penicillium chrysogenum* using flow cytometry—the fast alternative to microscopic image analysis

**DOI:** 10.1007/s00253-017-8475-2

**Published:** 2017-09-14

**Authors:** Daniela Ehgartner, Christoph Herwig, Jens Fricke

**Affiliations:** 1 CD Laboratory on Mechanistic and Physiological Methods for Improved Bioprocesses, TU Wien, Gumpendorferstrasse 1a/166, 1060 Vienna, Austria; 2 Research Area Biochemical Engineering, Institute for Chemical, Environmental and Biological Engineering, TU Wien, Gumpendorferstrasse 1a/166, 1060 Vienna, Austria

**Keywords:** Filamentous fungi, Flow cytometry, Morphology, Pellets, Microscopy, Image analysis, Hyphae

## Abstract

An important parameter in filamentous bioreactor cultivations is the morphology of the fungi, due to its interlink to productivity and its dependency on process conditions. Filamentous fungi show a large variety of morphological forms in submerged cultures. These range from dispersed hyphae, to interwoven mycelial aggregates, to denser hyphal aggregates, the so-called pellets. Depending on the objective function of the bioprocess, different characteristics of the morphology are favorable and need to be quantified accurately. The most common method to quantitatively characterize morphology is image analysis based on microscopy. This method is work intensive and time consuming. Therefore, we developed a faster, at-line applicable, alternative method based on flow cytometry. Within this contribution, this novel method is compared to microscopy for a penicillin production process. Both methods yielded in comparable distinction of morphological sub-populations and described their morphology in more detail. In addition to the appropriate quantification of size parameters and the description of the hyphal region around pellets, the flow cytometry method even revealed a novel compactness parameter for fungal pellets which is not accessible via light microscopy. Hence, the here presented flow cytometry method for morphological analysis is a fast and reliable alternative to common tools with some new insights in the pellet morphology, enabling at-line use in production environments.

**Electronic supplementary material:**

The online version of this article (10.1007/s00253-017-8475-2) contains supplementary material, which is available to authorized users.

## Introduction

An important parameter in filamentous bioreactor cultivations is the morphology of the fungi. Morphology and productivity are highly interlinked and depend on process conditions. Filamentous fungi exhibit a large variety of morphological forms in submerged culture. These forms range from dispersed hyphae, to interwoven mycelial aggregates, to denser hyphal aggregates, the so-called pellets. Depending on the aimed product, different characteristics of morphology are favorable (Papagianni 2004). Dispersed growth was described to achieve better production performance of glucoamylase (Gibbs et al. 2000), while pellets were related to citric acid production (Papagianni 2004). However, it is not just that the productivity is directly linked to the morphology, but also the process is affected. Several studies were conducted, investigating the connection of morphology and viscosity. The latter is linked to mass transfer and energy input (Chain et al. 1966; Petersen et al. 2008; Riley et al. 2000). As a general trend, it can be stated that filamentous growth with high amounts of hyphae causes increased viscosity (Quintanilla et al. 2015). Furthermore, high fraction of pellets results in better mass and heat transfer, and lower power input levels needed for mixing (Znidarsic and Pavko 2001). Various factors build a complex system of interactions. Operation conditions influence the growth, the product formation, and the morphology. In addition, filamentous growth directly influences the morphology, which, consequently, further changes the viscosity and which in turn has an impact on the operation conditions (Quintanilla et al. 2015).

In the recent decades, the investigation of the fungal morphology in submerged bioreactor cultures is a central issue. Microscopy in combination with image analysis is the most common method (Cox et al. 1998; Paul and Thomas 1998; Posch et al. 2012; Vanhoutte et al. 1995). Automated image recording and automated analysis of images allow a high-throughput, statistically verified morphological analysis (Posch et al. 2012). Furthermore, online methods for image analysis exist like for example the quantification of morphology in flowthrough cells. These last mentioned online analyses focused only on hyphal morphology. The flow cell with a height of 40 μm limits these tools to dispersed growing cultures (Christiansen et al. 1999; Spohr et al. 1998).

Most common morphological classifications distinguish between freely dispersed mycelia and aggregates. Freely dispersed mycelia include hyphae, which are long and can have branches (Cox et al. 1998). Simple clumps, also called small clumps or entanglements, are larger freely dispersed mycelia where the main hypha is not identifiable. These are often referred as “artificially overlapping hyphae” (Cox et al. 1998; Paul and Thomas 1998; Posch et al. 2012). A further dispersed morphological class is clumps, also called large clumps (Cox et al. 1998; Paul and Thomas 1998; Posch et al. 2012). These consist of aggregated or clumped hyphae (Cox et al. 1998). Large clumps are distinguished from so-called pellets by the missing of a dense core. Latter is a central dark region in the center of the aggregate, which is typical for pellets. The core is surrounded by a brighter outer mycelial region, the “hairy” annular region. Pellets have the size of several hundred micrometer to more than 1 ml (Cox et al. 1998; Paul and Thomas 1998). Cox et al. (1998) pointed out that pellets are three-dimensional, which possibly cannot be sufficiently covered by image analysis based on microscopy. As a more appropriate investigation, a chamber on the microscope stage to preserve the shape is proposed. Methods making pictures on microscope slides assume pellets to be nearly spherical (Cox et al. 1998). Various morphological parameters are evaluated concerning length/size/diameter of hyphae and hyphal aggregates. Morphological evaluation of pellets focuses apart from size evaluation, especially on the description of the annular area and the annular area compared to the core (Paul and Thomas 1998).

Although flow cytometry has often been applied for the morphological description of microorganisms as bacteria (Ehgartner et al. 2015; Langemann et al. 2016), investigations of filamentous organisms apart from the spore stadium (Ehgartner et al. 2016a, b) are scarce. Therefore, the main reason is the size limitation of common flow cytometers. Hyphal aggregates risk to clog the tubing (Dubelaar and Gerritzen 2000). Only large-particle flow cytometers ought to cope with pellets of several hundred micrometers diameter. So far, investigations of filamentous fungi in the pellet stadium focused on size, density, and fluorescence measurement, but did not go more into detail concerning the morphology (de Bekker et al. 2011; Delgado-Ramos et al. 2014). In addition, an application of image analysis for morphological investigation of pictures taken in the flow cell was recently published (Golabgir et al. 2015).

We aim to develop an at-line applicable high throughput morphology analysis method for filamentous fungi as a faster and easier applicable method to image analysis via microscopy. The method ought to be applicable for disperse morphology and pellet cultures. The detail of morphological classification and description should be comparable to image analysis via microscopy in terms of the range of measurement error. In this work, we present how morphological data was extracted, compare the results to the standard image analysis method developed by Posch et al. (2012), and show the application of morphological analysis in two fed-batch bioreactor cultivations.

## Materials and methods

### Strain

Spore suspensions of the P-14 *Penicillium chrysogenum* candidate strain for penicillin production descending from the P-2 *P. chrysogenum* candidate strain (American Type Culture Collection with the access number ATCC 48271) (Lein 1986) were kindly provided by Sandoz GmbH (Kundl, Austria) and used for all experiments.

### Bioreactor cultivations

Cultivations were performed in two Techfors S bioreactors (Infors HT, Bottmingen, Switzerland) with 10- and 20-l maximal working volume. The batch was cultivated with an initial volume of 6.5 l in the first mentioned bioreactor and inoculated with 2∙10^8^ spores/l. During batch phase, the pH was not controlled. The end of the batch was defined as an increase in the pH of 0.5 by convention. After the batch, the broth was diluted with fed-batch medium (15% broth, 85% medium) and two fed-batches were started in parallel with an initial volume of 6.5 and 13 l, respectively. Batch and fed-batch media were similar as described elsewhere (Posch and Herwig 2014).

During fed-batch phase, the pH was kept constant at 6.5 ± 0.1 by addition of 20% (*w*/*v*) KOH or 15% (*v*/*v*) H_2_SO_4_, respectively. The pH was measured using a pH probe (Hamilton, Bonaduz, Switzerland). After additional 12 h, the nitrogen and the phenoxyacetate feed were started. Ammonium and phenoxyacetate were fed at constant rates. In the first 48 h of fed-batch, 500-g/l glucose solution was fed at a constant rate of 1.01 ml/(l∙h). Afterwards, the feed was continued at the same level in fed-batch 1 (FB1), and an exponential ramp was started in fed-batch 2 (FB2) leading to a maximal feed rate of 4.1 ml/(l∙h) of the very 500-g/l glucose solution after 120 h of fed-batch. This maximal feed rate was maintained for the last 30 h of cultivation in FB2. Apart from these differences in the feeding strategy, FB1 and FB2 only differed in the size of the bioreactors and hence of the cultivation volume (6.5 vs. 13 l).

Three additional fed-batches were carried out for method development varying in spore inoculum concentration (2∙10^8^ spores/l vs. 2∙10^9^ spores/l), pO_2_ (< 10 vs. < 40%), and feeding strategy (constant feeding rate of constant rate of 1.01 ml/(l∙h) vs. constant feeding rate plus glucose pulse).

The stirrer was equipped with three six bladed Rushton turbine impellers, of which two were submersed and one was installed above the maximum liquid level for foam destruction. Fermentation temperature was kept at 25 °C via a cooling/heating jacket. The aeration was controlled at 1 vvm in batch and initial fed-batch with mass flow controllers (Vögtlin, Aesch, Switzerland). The dissolved oxygen concentration was measured using a dissolved oxygen probe (Hamilton, Bonaduz, Switzerland) and controlled between 40 and 90% during batch and between 40 and 60% during fed-batch. This control was conducted via adjustment of stirrer speed. The initial conditions were 325 rpm stirring speed in batch and 500 rpm in fed-batch. CO_2_ and O_2_ concentrations in the off gas were analyzed with an off-gas analyzer (M. Müller AG, Egg, Switzerland).

### Light microscopy and image analysis

Light microscopy imaging with subsequent automated image analysis was carried out based on the method developed by Posch et al. (2012).

Cultivation samples were diluted, so that the final biomass dry weight was around 1 g/l. Fifty microliters of Loeffler’s methylene blue per ml sample was added. Following, 50 μl of the stained sample was pipetted onto a standard glass slide (25 × 75 mm) and then covered with an extra-large cover slide (24 × 60 mm). The slide was then automatically scanned using a Leitz wide field microscope (Leitz, Stuttgart, Germany) with 63-fold magnification and an automated x-y-z stage (Märzhäuser, Wetzlar, Germany). Images were recorded with a five megapixel microscopy CCD color camera (DP25, Olympus, Tokio, Japan) and the image recording software analysis5 (Olympus, Tokio, Japan). A total of 450 images (1354.2 × 1015.65 μm) were recorded per slide, scanning two slides per sample.

Images were then automatically evaluated. Hyphal elements were thereby classified into unbranched hyphae, branched hyphae, small clumps, large clumps, and pellets. For all of these morphological classes, different size parameters were calculated. In addition, roughness and fullness (see Eq. 1 and Eq. 2) were determined for pellets. Further information including a list of evaluable morphological parameters, calculations, and statistical background can be found elsewhere (Golabgir et al. 2015; Posch et al. 2012).

1 $$ \mathrm{roughness}=\frac{{\mathrm{perimeter}}^2}{4\bullet \mathrm{Pi}\bullet \mathrm{area}} $$

Calculation of roughness due to Paul and Thomas (1998)

2 $$ \mathrm{fullness}=\frac{area}{\mathrm{convex}\  \mathrm{area}} $$

Calculation of fullness due to Paul and Thomas (1998)

### Flow cytometry

Samples from fed-batch were diluted 1:10 into phosphate-buffered saline (50 g/l of 2.65 g/l CaCl_2_ solution, 0.2 g/l KCl, 0.2 g/l KH_2_PO_4_, 0.1 g/l MgCl·6 H_2_O, 8 g/l NaCl, and 0.764 g/l Na_2_HPO_4_·2 H_2_O) and stained with propidium iodide (Sigma Aldrich, St. Louis, Missouri/USA; 20 mM stock dissolved in dimethyl sulfoxide (DMSO), diluted to a final concentration of 20 μM). After incubating 10 min, the sample was further stained with fluorescein diacetate (Sigma Aldrich, St. Louis, Missouri, USA; stock solution of 5 g/l dissolved in acetone) to a final concentration of 5 mg/l. After incubation of 1 min, the sample was further diluted (1:100 in the same buffer) for flow cytometric analysis. The sample pump speed was set to 15 μl/s and each measurement took between 60 and 120 s.

A CytoSense flow-cytometer (CytoBuoy, Woerden, Netherlands) with two forward scatter (FWS), one sideward scatter (SWS), and three fluorescence channels (yellow, orange, red) was used for single cell analysis. The implemented laser had a wavelength of 488 nm. The configuration of the filter set was 515–562 ± 5 nm for the green/yellow fluorescence channel (used for fluorescein diacetate) and 605–720 ± 5 nm for the red fluorescence channel (used for propidium iodide). Implemented in the device was a PixeLINK PL-B741 1.3MP monochrome camera (PixeLINK, Ottawa, Canada) for in-flow image acquisition. For data treatment, the software CytoClus (CytoBuoy, Woerden, Netherlands) and a custom-programmed Matlab 2016b script (MathWorks, Nattick, Massachusetts, USA) were used.

The CytoSense flow-cytometer is a device constructed for the measurement of particles up to a size of 1 mm width and an even higher length due to its alignment in the sheath flow. This maximal measurable particle size is delimited by the diameter of the tubing within the system. To minimize size exclusion effects, the sample inlet was enlarged by mounting a funnel with a maximal diameter larger than 5 mm.

The CytoSense flow-cytometer provides multiple data points per channel per particle. This signal or so-called pulse shape is achieved for both scatter channels as well as green, orange, and red fluorescence channels (Dubelaar and Gerritzen 2000). For exemplary pulse shapes, see Ehgartner et al. (2016a). These pulse shapes are the basis for multiple curve parameters. The length of this pulse shapes is shown by the length parameters and represents the maximal diameter of the particle. Except for length parameters in μm, all parameters are in arbitrary units. The most relevant for the here presented study are the following:

- Maximum: Maximum of signal curve
- Total: Area beneath the curve
- Length: Length of the signal
- Sample length: Length of signal above trigger level
- Fill factor: Similarity of the curve to a block (0–1; the more block-shaped, the higher)

Furthermore, the image-in-flow feature enables picture taking of particles in the flow cell. These pictures are connected to measurement data of the respective particle, facilitating method development by the visual evaluation of the measured particle.

## Results

### Differentiation of morphological classes via clustering

To differentiate morphological classes like the ones described in the introduction section, visual and statistical clustering was applied based on curve properties of SWS and FWS signals. The investigation was based on FWS and SWS which represent size, shape, and surface properties of measured elements (Dubelaar and Gerritzen 2000).

For the distinction of fungal elements from media background, only elements with a threshold of total green fluorescence higher than 30 representing fluorescein diacetate staining were evaluated. In a first step, scatter plots were created and gates for morphological classification were set to represent observed clusters. Therefore, “natural” clusters found in the scatter plots were investigated concerning their morphological commonalities. The image-in-flow feature supported the identification of the morphological classes. This was done by the visual evaluation of the picture and the classification into single hyphae, rather loose aggregates or pellets with a dense core. Morphological classification was based on the historical distinction of hyphae, small clumps, large clumps, and pellets which were introduced in the introduction section and are described more in detail elsewhere (Cox et al. 1998; Paul and Thomas 1998). An example for gate setting is presented in Fig. S1 in the supplementary material.

For more exact setting of the gate boundaries defined by this visual clustering, cluster analyses were calculated in Matlab (MathWorks, Nattick, Massachusetts, USA). Only analyses resulting in up to five clusters were then further investigated. Some clusters found by cluster analysis were totally different than the clusters found via visual clustering. Differences in the morphology between these clusters could not be enabled watching the pictures taken in the flow cell. Nevertheless, some of the statistical clusters were equivalent to the clusters found by visual clustering. These clusters from statistical clustering were helpful for adapting the boundaries of the gates set via visual clustering (see Table 1).

**Table 1:** Cluster analyses and morphological classes distinguished

Cluster analysis	No. of clusters	Clusters equivalent to visual clustering
k-means	4	1 cluster represented all hyphal aggregates
k-means	3	Pellets, large clumps
Hierarchical average Euclidean	2	Small elements vs. large elements

### Definition of morphological classes

**Fig. 1:**
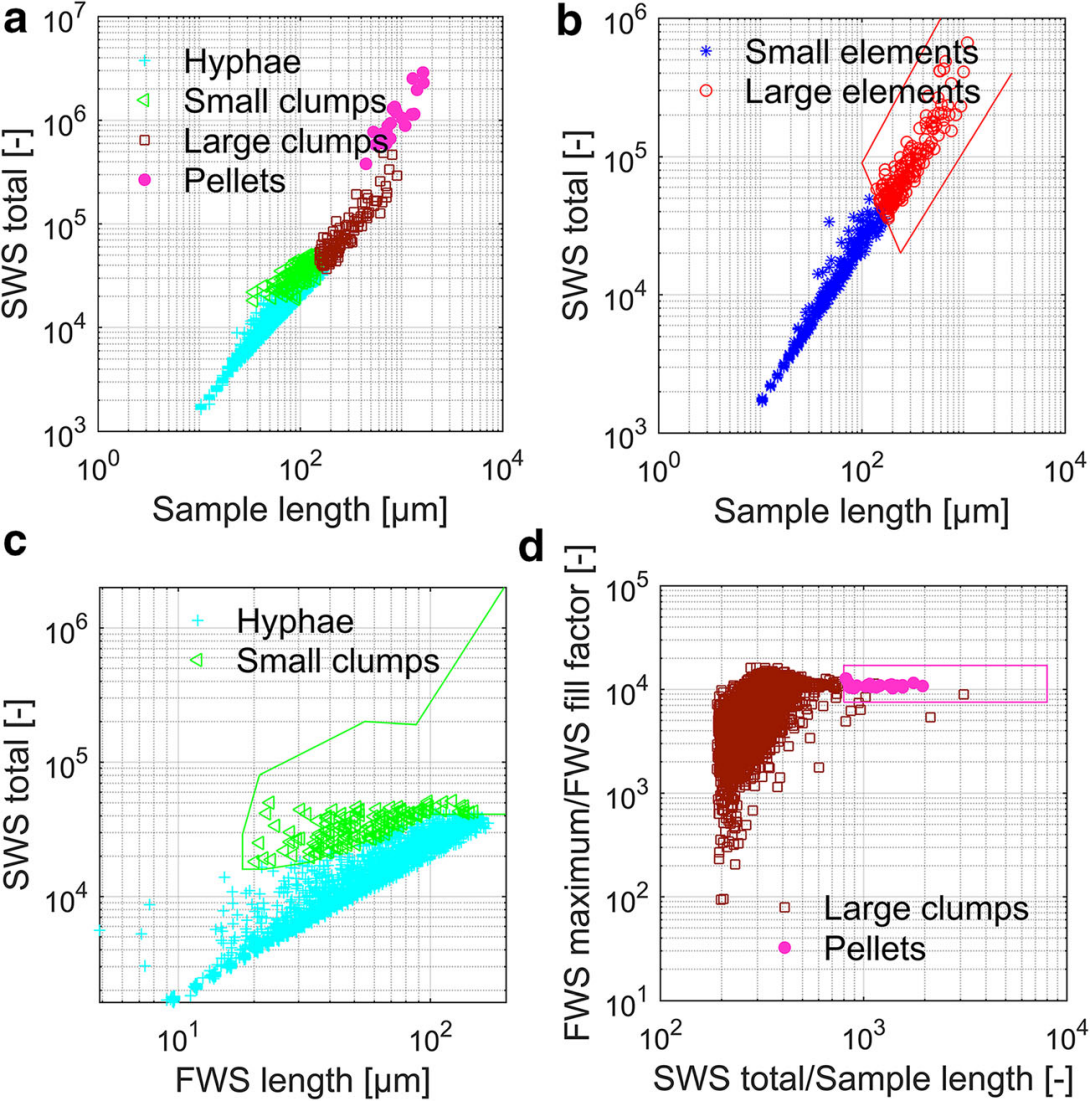
Distinction of morphological classes. **a** All four morphological classes. **b** Small elements including single hyphae and small clumps are represented as blue stars while large elements consisting of large clumps and pellets are in the red gate shown as red circles. These two populations are distinguished by sample length and SWS total. **c** Further investigation of small elements: FWS length and SWS total distinguish hyphae (turquoise crosses) and small clumps (green triangles). Elements placed in the green gate are small clumps. Small elements outside of this gate were defined to be hyphae. **d** Further investigation of large elements: One distinction criteria for pellets from large clumps was by SWS total/sample length and FWS maximum/FWS fill factor. A large element found in the pink square-like gate which in addition has a saturated FWS was defined to be a pellet (see pink dots). Large elements not meeting one or both criteria are large clumps which are represented by dark red squares

The thereby extracted differentiation of morphological classes is shown in Fig. 1. Figure 1a shows all morphological classes in one plot. It underlines that the morphological classes are mainly differentiated by size and shape. Still, it is not possible to distinguish all classes with only two scatter parameters. Summarizing the scatter plots in Fig. 1b–d, the following values are needed to distinguish the four sub-classes hyphae, small clumps, large clumps, and pellets:

- Small vs. large elements: sample length vs. SWS total (Fig. 1b)
  - Further differentiation of small elements ➔ hyphae vs. small clumps: FWS length vs. SWS total (Fig. 1c)
  - Further differentiation of large elements ➔ large clumps vs. pellets: SWS total/sample length vs. FWS maximal/FWS fill factor (Fig. 1d)

A further distinction of pellets from large clumps apart from the properties depicted in Fig. 1d is that pellets are defined to have a dense core. This is reflected in a saturation in the FWS signal (FWS maximum = 10^4^).

### Comparing flow cytometry and microcopy method

#### Definition of morphological classes

In Table 2, the definitions of the morphological classes as described above are compared to the morphological classes of image analysis developed by Posch et al. (2012).

**Table 2:** Definition of morphological classes for the flow cytometry method vs. image analysis

	Microscopy	Flow cytometry
Hyphae	• Area bigger than 100 μm^2^: → When a hyphal diameter of 3 μm is assumed, this means that the minimal length of hyphae is 33.3 μm. • Area smaller than 3500 μm^2^ • Skeletonized pictures do not form a loop (Paul and Thomas 1998)	• Sample length between 10 and 150 μm • SWS total smaller than 4∙10^4^ • FWS length below 190 μm → Exact ratios of SWS total and FWS length see in Fig.1c
Small clumps	• Area in-between 1000 and 3500 μm^2^ • Skeletonized pictures form a loop (Paul and Thomas 1998).	• Sample length between 10 and 150 μm • SWS total in-between 1.8∙10^4^ and 9∙10^4^ • FWS length higher than 18 μm → Exact ratios of SWS total and FWS length see in Fig. 1c.
Large clumps	• Area bigger than 3500 μm^2^ • Hyphal aggregate not classified as pellet	• SWS total higher than 2∙10^4^ and sample length bigger than 100 μm • Is not a pellet
Pellet	• Area bigger than 7500 μm^2^ • Has a core • Core area bigger than 7000 μm^2^	• SWS total higher than 4∙10^4^ and sample length bigger than 120 μm • A core exists (saturated FWS) • Ratio of FWS maximal: FWS fill factor bigger than 7.5∙10^3^ • SWS total:sample length bigger than 8∙10^2^

The ratio of the length parameter compared to SWS total represents the proportion of the maximal length of the hyphal element compared to the total size of the element represented by the SWS total. While the distinction of hyphae and small clumps as well as of small and large elements is clearly based on size and shape, the definition of pellets except from the saturated FWS seems more complex. Both, FWS maximal/FWS fill factor and SWS total/sample length, give a more-dimensional representation of the measured element. FWS maximal/FWS fill factor combines size (maximum of signal curve) with three-dimensional shape properties given by the fill factor. Similar is the case for SWS total/sample length. Here, the ratio of three-dimensional size (area beneath the curve being influenced by the length of the element but also by height and surface) to the one-dimensional size (length of element) is presented.

#### Element sizes and total size distribution

**Fig. 2:**
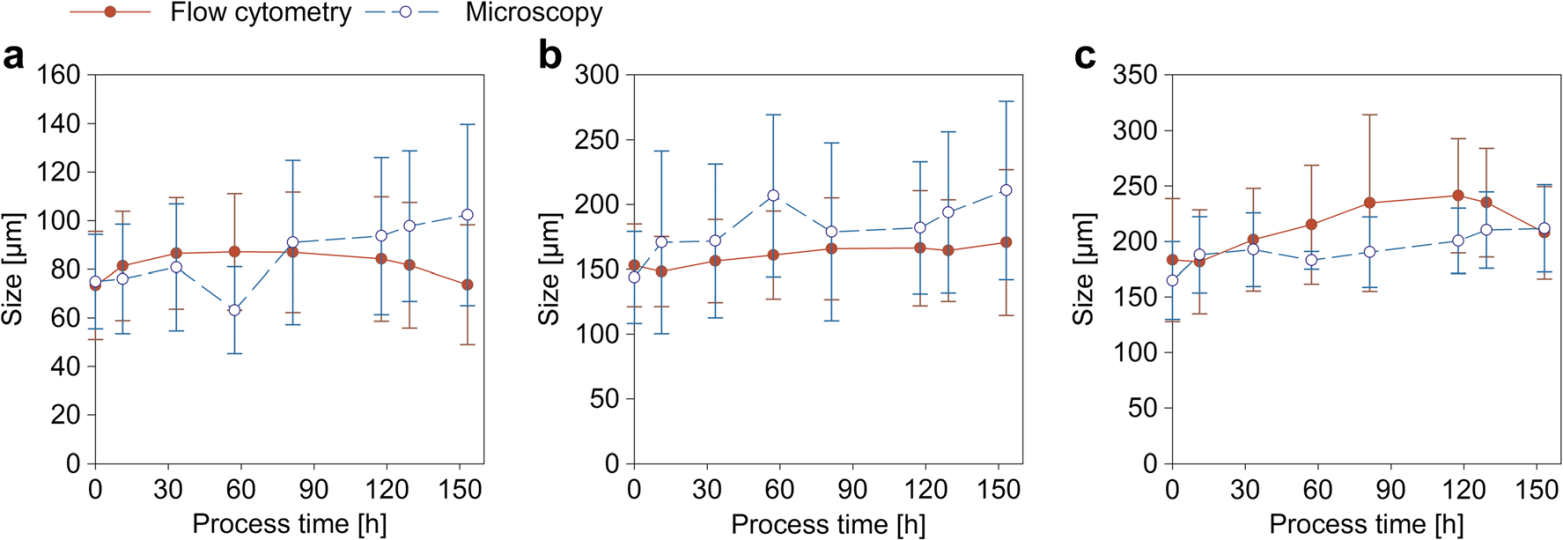
Average size of small clumps (**a**), large clumps (**b**), and pellets (**c**) over process time of FB1 determined by flow cytometry and the microscopy method. For the representation of size, the parameter used in flow cytometry was sample length, while the length of hyphae respective the diameter of aggregates is shown for microscopy. The error bars show the size distribution for each class

Figure 2 shows average size and size distribution over process time determined by both methods (microscopy and flow cytometry) for small clumps (a), large clumps (b), and pellets (c). Although the sizes are overlapping, clumps tend to be smaller in flow cytometry, while pellets are in average slightly larger. These differences are caused by variations in the definition of morphological classes between the two methods. However, the average size of the morphological classes is for both methods in the same dimension. This is an important finding as the methodology for morphological description via flow cytometry differs from microscopy, but still, the definition of the morphological sub-classes ought to be equivalent. Hence, the flow cytometry method does not aim to invent new morphological sub-classes but stresses much more to yield well-known classification into hyphae, clumps, and pellets and its statistically consistent quantification.

**Fig. 3:**
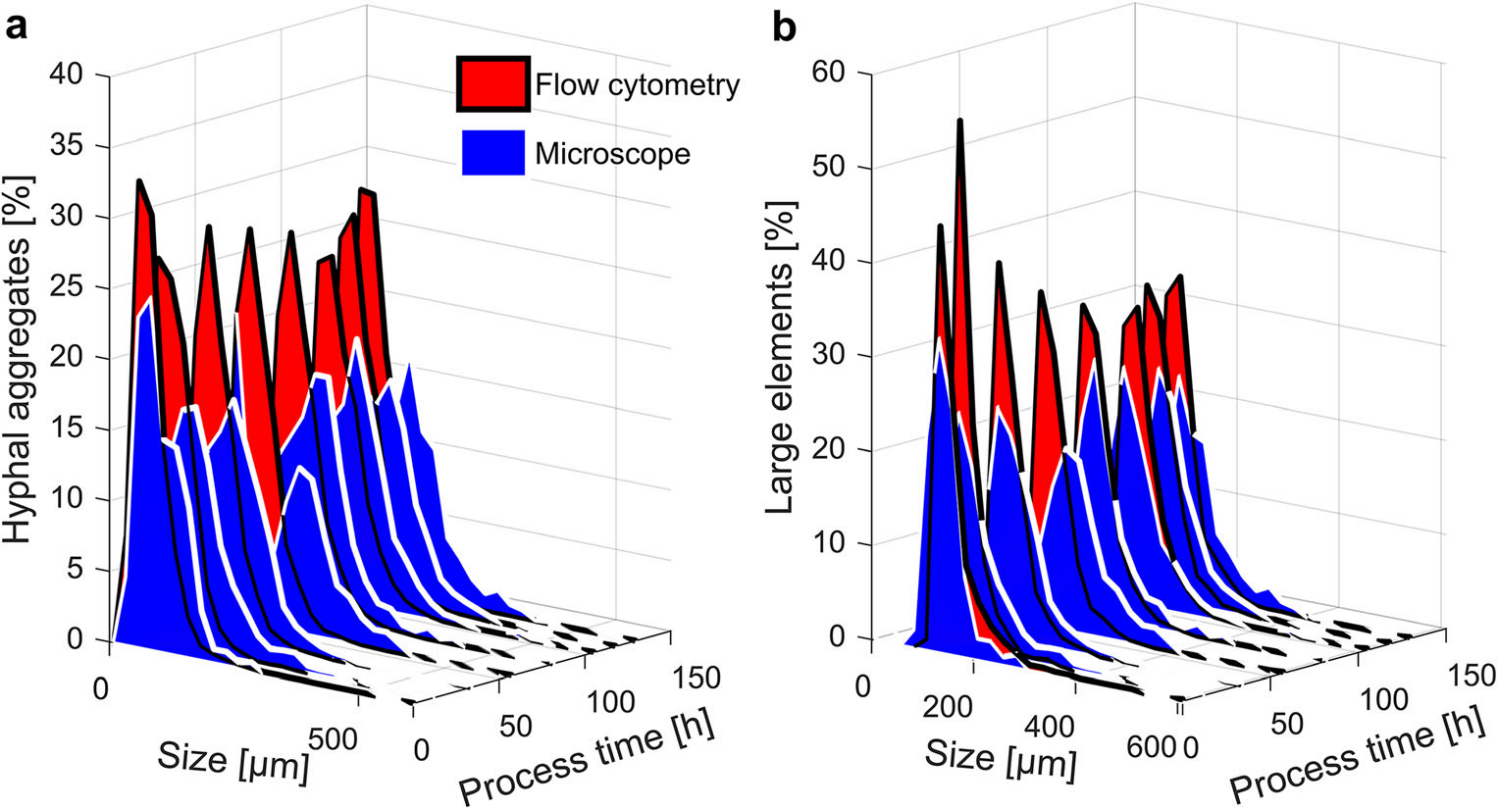
Size distribution over process time determined via flow cytometry and the microscopy method for FB1. **a** Hyphal aggregates, **b** large elements (large clumps and pellets)

Figure 3 shows that size distributions of hyphal aggregates and large elements resulting from both methods are in the same range. Microscopy reveals in average slightly bigger aggregates and large elements than flow cytometry. The size distributions are presented in relative values related to the total number of hyphal aggregates (Fig. 3a) respective large elements (Fig. 3a) measured with each of the two methods.

#### Pellet morphology

**Fig. 4:**
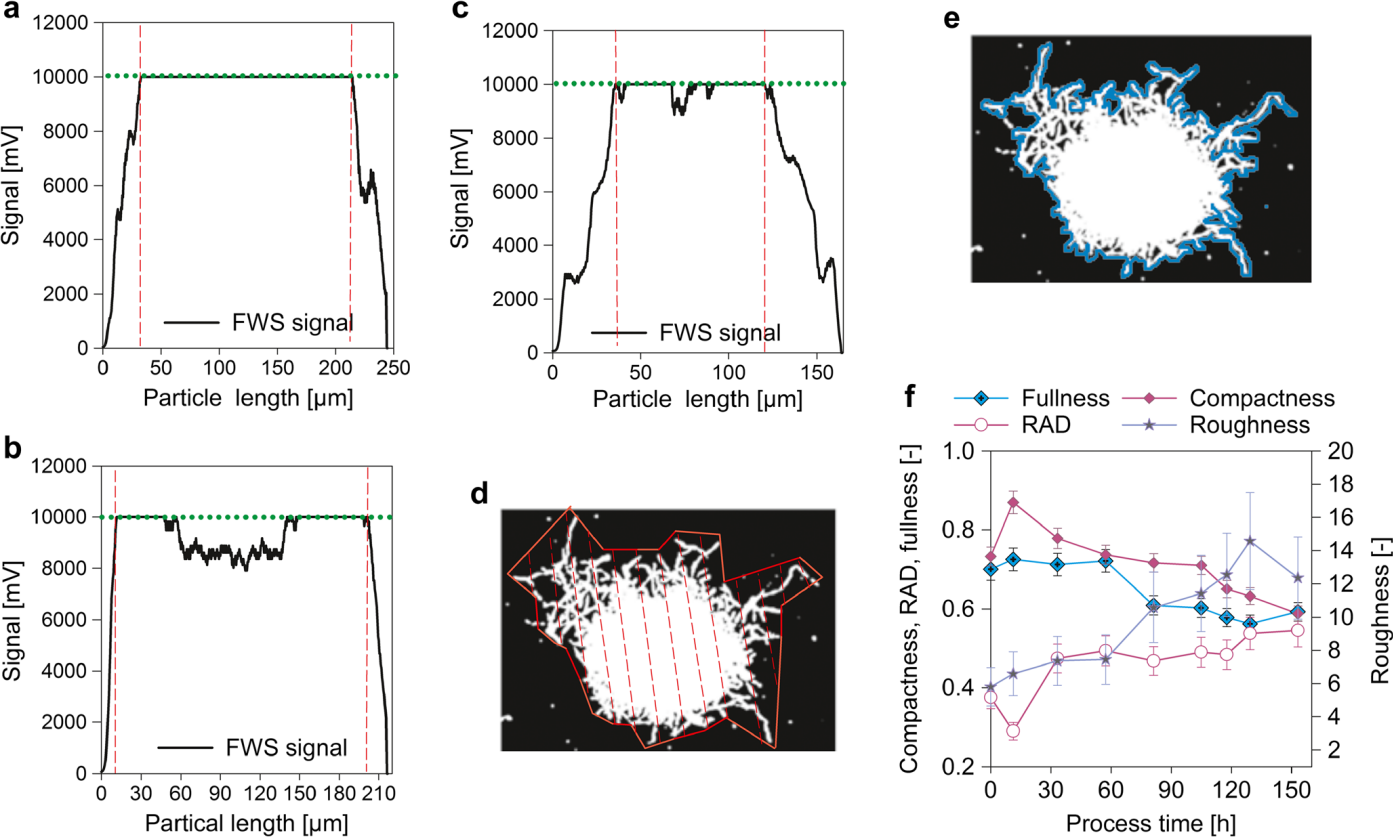
Fullness and roughness from the microscopy method and corresponding parameters deriving from the FWS signal of the flow cytometer. **a**–**c** The FWS signals of three pellets are shown. The inner pellet core is indicated by two vertical lines. The horizontal line shows the saturation of the FWS signal. **a** Pellet with low relative annular diameter (0.26) and high compactness (1.0). **b** Pellet with very low relative annular diameter (0.12) and low core compactness (0.49). **c** Pellet with high relative annular diameter (0.46) and average core compactness (0.77). **d**, **e** Pellet pictures from microscopy after conversion to a binary image. The convex area for fullness calculation (**d**) and the perimeter for roughness calculation (**e**) are shown. The area for calculation of these to parameters is the sum of all white pixels of the pellet. **f** Comparison of RAD determined via flow cytometry and pellet fullness and roughness measured with microscopy for FB2. In addition, core compactness is shown. Pictures of the pellets shown in Fig. 4 **a–c** can be found in Fig. S2 in the supplementary material

Signal properties can be used to describe single morphological classes more in detail, as for example pellets. Fullness and roughness are parameters resulting from light microscopy comparing pellet core and the less dense hyphal area in the outer layer. For more details of calculation, see Eq. 1 and Eq. 2. The parameter “relative annular diameter” (RAD) (see Eq. 3) was set up for the flow cytometry method, to reveal a parameter with similar information about pellet morphology. The value of RAD decreases when the layer of hyphae at the outside of the pellet is smaller (see Fig. 4a–c, Fig. S2 in the supplementary material). Plotting RAD over process time together with the pellet fullness and the pellet roughness (see Fig. 4f) shows the similarity of the information.

3 $$ \mathrm{RAD}=\frac{\mathrm{Annular}\  \mathrm{diameter}}{\mathrm{Particle}\  \mathrm{length}} $$

Relative annular diameter (RAD). The annular diameter is the length of the annular area (outside of pellet core—see Fig. 4a–c). The particle length is the length of the FWS signal as shown in Fig. 4a–c

By considering the definitions of morphological classes for the flow cytometry and the image analysis, differences in the time courses of the pellet specific parameters are self-evident. While the roughness is based on changes of the real perimeter respective to the perimeter of a circle of the same area, RAD calculation is based on the ratio of the diameter of the annular region and the diameter of the whole pellet. The annular diameter is the length of FWS from the beginning of the signal until the first saturation of the signal is reached, and this from both sides of the pulse shape. However, a FWS signal below saturation does not necessarily represent loose hyphal structure, but may as well represent degraded core regions as shown in Fig. 4b. The FWS signal in the center of this pellet is not saturated. This region of the pellet would be supposed to be the “weak point” of the pellet, the region where it may break at high shear forces. After breakage of such a pellet, this region would be counted as annular area, as it is between the start of the FWS signal and its first saturation. Thus, such a broken pellet has a high RAD, while the roughness determined by microscopy would be proportionally low. Latter is the case because this vacuolized/lysed region seems black/dark on the picture as a normally dense core does. Hence, it is not visible under the microscope (see Fig. S2b in the supplementary material).

A weak point, which both methods for annular region description—roughness determination via microscopy and RAD via flow cytometry—have in common, is that these values represent a two-dimensional measurement of a three-dimensional phenomenon. In large pellets, the core may seem bigger than it really is, as hyphae from the annular region are pressed together and hence seem dark when put on the microcopy slide. Similarly, it may occur in flow cytometry. In larger pellets, the light needs to pass through more hyphae in the annular region to reach the detector because of the higher pellet diameter. Therefore, the FWS signal may easily be saturated than in pellets with lower diameter.

Another parameter based on the pulse shape of FWS is the pellet core compactness (short “compactness”). As Fig. 4a–c and Eq. 4 show, this parameter represents the density of the pellet core. Older hyphae are described to contain high numbers of vacuoles, which makes them weak and more possible to break (Paul et al. 1998). The here presented compactness value decreases with the age of the pellets (Eq. 4f), possibly due to vacuolization and lysis.

4 $$ \mathrm{Compactness}=\frac{\mathrm{Saturated}\ \mathrm{FWS}\ \mathrm{signal}\  \mathrm{in}\  \mathrm{pellet}\  \mathrm{core}}{\mathrm{Core}\  \mathrm{diameter}} $$

Compactness of the pellet core. This parameter is calculated as the relative length of saturated FWS signal in the pellet core

As the RAD does not distinguish between young rough pellets and broken degraded ones, the compactness can be used as additional parameter for the discrimination of those. A pellet with high RAD and low compactness is more likely to be a degraded one than a young rough pellet, where the pellet core is still intact. Furthermore, RAD and compactness over passage of time give insight into the cause of high RAD values.

#### Measurement error and robustness

As stated in the introduction section, we aim to present an at-line applicable high throughput method for morphological analysis which is faster, but comparable to state-of-the-art image analysis via microscopy. To be a reasonable alternative, the robustness and validity of the method are of utter importance.

By taking 900 pictures per sample with the image analysis method, approximately a volume of 0.5 to 2.5 μl is analyzed. In detail, two microscopy slides with 50 μl of 1:10 to 1:50 diluted broth per slide are investigated. The dilution depends on the biomass concentration, as the samples ought to be diluted to a final biomass concentration beneath 1 g/l to avoid overlapping of hyphal elements. Compared to the volume of 0.5 to 2.5 μl investigated via microscopy, approximately 5 μl of the original sample is investigated with the flow cytometry method. This is 2- to 10-fold more investigated sample. In flow cytometry, each sample is measured at least four times in a 1:1000 dilution. In one measurement, in average, 1200 μl of the 1:1000 diluted sample is measured. Thus, four measurements per sample result in a volume of approximately 5 μl undiluted sample investigated with the flow cytometer.

The average errors of the two methods are shown in Table 3. Errors were evaluated using three samples, taken at different process times. The samples varied in various parameters as biomass concentration (10–25 g/l), pellet fraction (13–23%), and SWS length of pellets (182–237 μm). For microscopy, six slides were measured per sample to evaluate the error. In flow cytometry, a 4-fold measurement was applied for error calculation.

**Table 3:** Measurement errors for the most common morphological parameters of microscopy and flow cytometry

	Microscopy		Flow cytometry	
	%	Absolute	%	Absolute
Diameter/SWS sample length small clumps	5	4 μm	1	1 μm
Diameter/SWS sample length large clumps	4	7 μm	1	2 μm
Diameter/SWS sample length pellets	6	11 μm	4	9 μm
Roughness/fullness pellets	20/4	1.6/0.03	–	
Compactness pellets	–		3	0.02
Relative annular diameter pellets	–		8	0.03
Conc./ml hyphae	–		7	58∙10^4^ hyphae/ml
Conc./ml small clumps	–		4	61,000 small clumps/ml
Conc./ml large clumps	–		10	39,000 large clumps/ml
Conc./ml pellets	–		24	7500 pellets/ml

The average measurement errors are given in % and in absolute units

Only few parameters can be compared, as the microscopy method does not allow the evaluation of absolute concentrations. Image analysis based on microscopy is no method resulting in concentration measurements. All data received are relative values compared to the measured population. A big part of the microscope slide is scanned, but nevertheless, there are regions not measured. In addition, excess fluid at the border of microscope slides is discarded. Hence, an exact calculation of the measured concentration of hyphal aggregates is not possible.

Concerning the determination of the size of pellets, the error is comparable (6 vs. 4%). The error for the diameter investigation of clumps is lower for the flow cytometry method (1%) compared to microscopy (4–5%). Errors of the microscopy method could be decreased by evaluating higher amounts of pictures (e.g., 4000 instead of 900) which was done when originally published (Posch et al. 2012). But taking four times more pictures than it was done for this study would increase the workload for morphological investigation by far and is thus not applicable for using it as real-time method.

Concerning the description of pellet morphology, the most robust determination was the evaluation of fullness (4%). The pendant of the flow cytometry method—RAD—showed an average error of 8%. The high variation (20%) for roughness determination can be explained by the high dependency of perimeter calculation on the conversion of the original picture to a binary one. Dependent on the evenness of the Köhler illumination and the contrast during microscopy, the quality of the microscopic pictures, especially of the hyphae, varies. For the conversion to a binary image, a threshold level has to be set. The difficulty thereby is to adjust the threshold in that way, which all hyphae are fully recognized as part of the hyphal aggregate, but that shadows are not recognized as such. Hence, it is a factor of variation, especially when the pictures are taken on different days leading to slightly different adjustments of the microscope.

The parameters not accessible via image analysis but measurable with the flow cytometry method are compactness and the concentration of hyphae and hyphal aggregates as shown in Fig. 4. For compactness, the average error was 3%, which is by far lower as for example the error on RAD (8%). Concerning the concentration of the morphological classes, the error increases with the decrease of prevalence of representations of each class. For illustration, in the sample at *t* = 80 h of FB1 3∙10^4^ pellets/ml were measured. Twenty times more large clumps were found. Numbers for hyphae and small clumps were above 10^6^ per milliliter sample. Hence, the number of pellets in the 1:1000 diluted sample for flow cytometry is extremely low—especially when compared to hyphae and small clumps.

### Application of flow cytometry method: morphological description of two fed-batches

#### Morphological classes

In the following, two fed-batches are analyzed with the here presented flow cytometry method. The fed-batches derived from one batch and differed in their feeding profiles. While FB1 had a constant feed throughout the whole process, FB2 was fed with an exponential profile starting after 48 h.

**Fig. 5:**
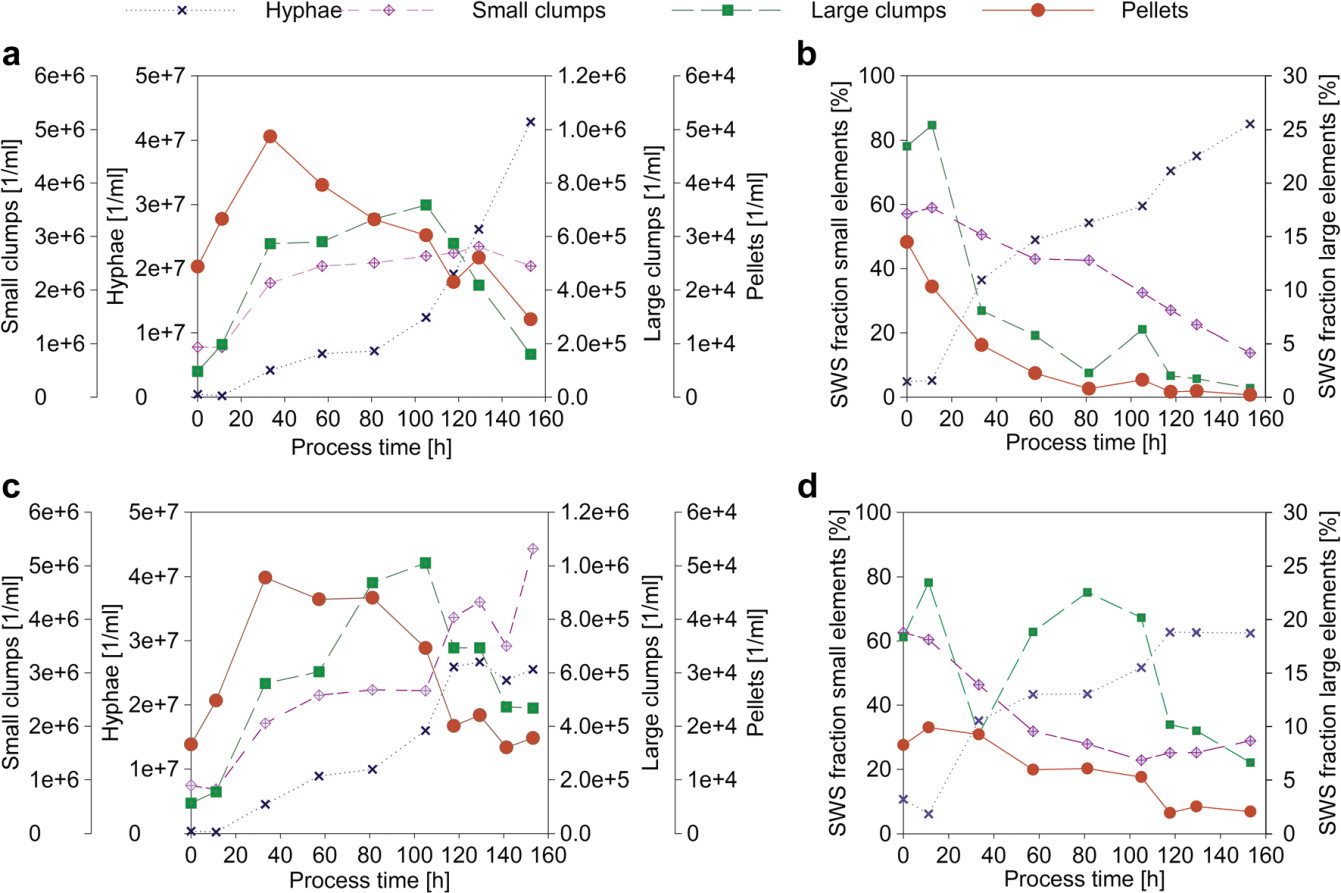
Distribution of the hyphal elements in the different morphological classes for FB1 (**a**, **b**) and FB2 (**c**, **d**). **a**, **c** Absolute concentrations of hyphae and hyphal aggregates differentiated into morphological classes. **b**, **d** SWS fractions of the morphological classes

The concentration of elements divided into morphological classes is shown in Fig. 5a, c. An alternative representation of the morphological classes is the SWS fraction (see Eq. 5/Fig. 5b, d).

5 $$ SWS\  fraction={\frac{\sum SWS\ {total}_{Elements class\ x}}{\sum SWS\ {total}_{AllElements}}}^{\ast }100 $$

#### SWS fraction

The SWS fraction explains the ratio between the SWS total values of an elemental class to the total value. With this fraction, the distribution of fungal biomass in morphological classes could be better shown than with the specific concentration of the elements. This is explainable by the enormous differences in volume of one single hyphae compared to one single pellet. Therefore, the SWS fraction ought to be more feasible to represent the distribution of biomass volume in the morphological classes. However, for direct comparison of fed-batches, the concentration of elements could be of advantage as a direct measurement is available.

An alternative to SWS fraction could be FWS fraction, as FWS is better known to represent size. However, FWS is saturated when pellets are measured. Thus, the number of pellets would be underestimated.

Comparing the concentration of hyphae and the concentration of hyphal aggregates for FB1 and FB2 (shown in Fig. 5a, c) results in the following insights: in the first 40 h, nearly identical trends for the fed-batches were observable. Only the concentration of pellets in FB1 was slightly higher. Concentrations in all morphological sub-classes were raising for the first 40 h of the fed-batch. As both fed-batches were inoculated from the same batch and had the same feeding profile during the first 48 h, the same trends and concentrations of morphological sub-classes were expected. Slight differences, especially concerning the pellet concentration, may result from sedimentation effects during the transfer of the batch culture to the fed-batch medium.

After 48 h, the feed in FB2 began to raise exponentially, while the feed in FB1 was held constant. This difference in the feeding profile is reflected in morphological differences. The pellet concentration in FB1 decreased sharply while the concentrations of clumps raised slowly. Further, the concentration of hyphae increased almost exponentially to reach a maximum at the end of the cultivation. In FB2, the pellet concentration was constant until *t* = 80 h. In addition, the concentration of large clumps increased until *t* = 105 h, and afterwards, the concentration of small clumps followed the same trend. The concentration of hyphae increased throughout the fed-batch. Still, the final concentration was only approximately the half of the more than 4∙10^7^ hyphae/ml in FB1.

The SWS fraction, shown in Fig. 5b, d, described a partially different situation. In FB1, a tendency to more large elements was observed. There were not only more pellets, but also the SWS fraction of large clumps was higher. As the concentrations of large clumps showed to be the same in Fig. 5a, c, this could be explained by the size of the single elements. In FB1 with the constant low feed rate, aggregates diminish in favor of free dispersed growth. Contrary, FB2 shows an increase in larger aggregates, especially until 80 h of process time. The largest contrast due to different feeding profiles was observed for large clumps. While the concentration constantly decreased in FB1, it strongly raised in FB2. At t = 80 h, the SWS fraction of large clumps in FB1 was 2% compared to 23% in FB2.

#### Size distribution

**Fig. 6:**
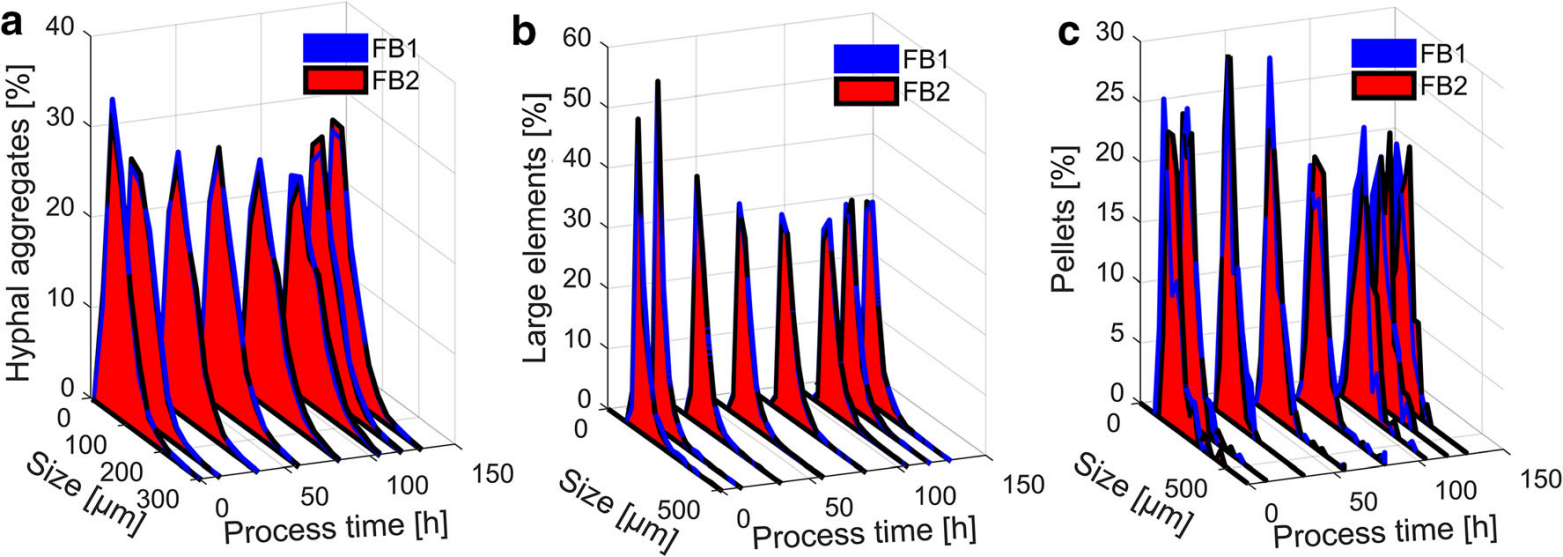
Comparing size distribution over process time for FB1 and FB2. Shown is the distribution of aggregates including small clumps, large clumps, and pellets (**a**), large elements including large clumps and pellets (**b**), and pellets only (**c**)

Figure 6 compares the size distributions of FB1 and FB2. Concerning hyphal aggregates and large elements, the size distribution did not differ significantly between the two cultivations. As a trend, the size distribution of hyphal aggregates became wider to reach a maximum at approximately 100 h. Further on, the wideness of the distribution decreased. The trend for large elements was different. Size distribution became wider over the whole process time.

#### Pellet morphology

**Fig. 7:**
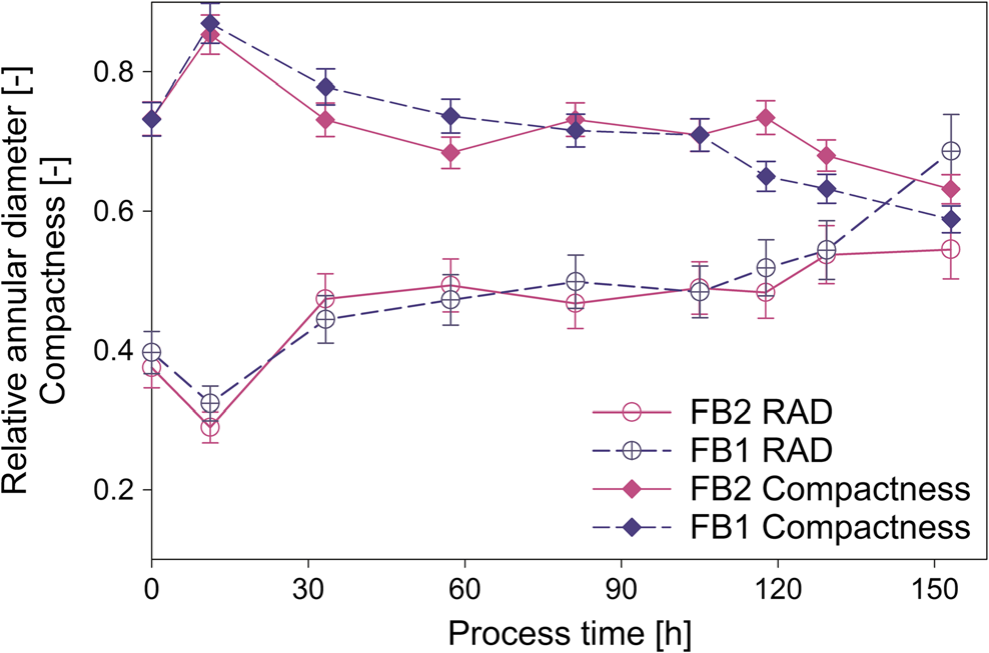
Relative annular diameter and compactness of pellets in FB1 and FB2

As already described above, RAD was introduced to describe the relative roughness/hairiness and the percentage of the annular area of the pellet. Figure 7 compares RAD for FB1 and FB2 over process time. Apart from the last sample, the time courses of RAD were comparable in both cultivations. The value decreased in the first 12 h of fed-batch, probably due to pellet formation and the growth to denser hyphal aggregate. After re-increasing until 40 h of the fed-batch, it stayed constant for 80 h. In the last 30 h of the fed-batch, it decreased in both cultivations, but to a higher extent in FB1.

Figure 7 compares the compactness for FB1 and FB2 over process time. In sum, the trend of compactness was opposed to the one of RAD. In FB1, the value decreased with ongoing process time. Opposed to that, it was constant in FB2 between 60 and 120 h. Then, it further decreased at the end of the cultivation. In the last hours of fed-batch, the compactness of FB2 was slightly lower than in FB1. This difference could probably be due to higher substrate limitation in the lower feeding regime of FB1.

The high increase of RAD at the end of FB1 (after 150 h) could be connected to the decrease of pellet compactness and ongoing pellet breakage. This goes in line with the sharp decrease of pellet and large clump concentration at the end of FB1 as shown in Fig. 5.

## Discussion

### Comparability of the methods

Within this contribution, a novel method for morphological analysis based on flow cytometry was presented. It aimed to be a faster alternative to image analysis via microscopy and was thus compared to the image analysis method described by Posch et al. (2012). In general, the following question should be answered: Are these two methods comparable? The morphological principle based on the flow cytometry method as well as image analysis is the same. Both methodologies differentiate several classes of morphology, depending on their size and grade of aggregation. Historically, this was the basis for several morphological analysis methods (Cox et al. 1998; Paul and Thomas 1998; Posch et al. 2012; Quintanilla et al. 2015). Both methods are applicable for filamentous cultures with a mixture of dispersed and pellet growth, but could also be applied for pure pellet cultures. The morphological parameters gained from the here presented method focus on size parameters for all morphological classes and the more detailed description of pellet morphology. Apart from the pellet compactness, which is not accessible as such by microscopy, all parameters fitted well between the two methods or showed the same trends in time courses. The occurrence of partially larger elements in the size distribution for the image analysis method can be explained by differences in the measurement principle. Hyphal aggregates ought to overlap on microscopy slides. Due to the branched morphology, hyphae and aggregates tend to intercalate, which makes differentiation on microscopic slides difficult. The sheath flow in the flow cytometer hampers overlapping of separate hyphae/hyphal aggregates.

Not comparable is the information about hyphae and hyphal aggregate concentrations. Image analysis per se is not a fully quantitative method, but results in relative distributions within the measured population. Hence, no absolute concentrations can be evaluated. Of course, rough estimations about absolute concentrations can be done, but these ought to be quite error prone. Quantitative data about the distribution of biomass within the morphological subclasses in image analysis are gained by the so-called area fractions. This parameter results from summing the area of all hyphal elements and relating the summed area of each sub-class to the total area (for more details, see Golabgir et al. (2015))). Thereby, the relative area of the sub-classes can be compared. But this method is not directly comparable to a concentration measurement, as a pellet takes more area in relation to hyphae. A similar approach to area fraction is the here presented SWS fraction. But the principle behind the two methods—area vs. scatter signal curve—is too different to try comparisons.

Another issue of comparability is the method of elimination of background as for example media particles. In image analysis, this is done via shape and fullness measurements (Paul and Thomas 1998). For the here presented flow cytometry method, a viability staining with fluorescein diacetate was conducted. All particles with green fluorescence were concluded to be hyphae and hyphal aggregates. Investigation of fluorescein diacetate staining with fluorescence microscopy had revealed that medium particles from our culture were not stained by this marker (Ehgartner et al. 2016b). Only some small hyphae were red fluorescent without showing green fluorescence. Although pellets showed to include dead biomass in the core, sufficient green fluorescence was present to be above the threshold and hence being classified as viable hyphal element. Consequently, a comparability of the flow cytometry method with image analysis should not be hampered by the methodology of background separation.

### Advantages and disadvantages of flow cytometry method

The here developed flow cytometry method for morphological analysis is able to describe pellet morphology. In addition, it produces information about the size of other morphological subclasses. So far, these other morphological subclasses were not further described by the here developed method. Hence, detailed description of freely dispersed cultures concerning branching grade, hyphal growth unit, and vacuolization grade as it was shown via image analysis methods is not (yet) possible (Cox et al. 1998; Paul and Thomas 1998; Posch et al. 2012; Vanhoutte et al. 1995). Description of hyphal tips as it is done via hyphal growth unit is possibly easier via microscopy, as hyphae are well represented on a two-dimensional space like a microscope slide. Approaching hyphal morphology via light scattering might be more abstract, but could be possible. The easy applicability of fluorescence dyes in flow cytometry promises to be a big advantage compared to image analysis. Via fluorescence staining, information about vacuolization and the evaluation of other physiological states as it was shown by Vanhoutte et al. (1995) is accessible. Evaluation of physiological states via fluorescence microscopy is possible, but automation of this microscopy method and development of an automated image analysis is even more difficult and time consuming than image analysis of light microscopy. In contrast, in flow cytometry, fluorescence staining is easily combinable with morphological analysis. Although the fluorescence was not further investigated, a viability staining combination was applied in this study. So far, it was only used to distinguish hyphal elements from background. An example would be the staining and herewith the localization of vacuoles via flow cytometry in hyphae or substrate diffusion in pellets. A similar approach was presented for the red fluorescence of chlorophyll in phytoplankton (Dubelaar et al. 2004).

Further advantages of the flow cytometer method are—as already mentioned—the measurement of hyphal elements in a liquid flow. Thereby, the overlap of hyphal elements is avoided.

The method is faster, even at-line applicable, and offers higher throughput than microscopy. In the way, the method was conducted for this study, at-line application is even crucial. The reason for this is the application of viability staining for the distinction of hyphal elements from background. Hence, offline measurements at a later stage are limited.

The higher throughput makes an investigation of more sample volume possible, giving a higher robustness to the method. This is shown by the comparison of the average measurement error between image analysis and the flow cytometry method. Latter tended to have a lower error. Another advantage in respect to lower measurement errors is the influence of the operator. Preparation of microscopic slides (regular distribution of hyphal element on the slide), adjusting of illumination and contrast, as well as threshold setting for binarization of images, is a matter of routine and partially operator dependent. These manual manipulations of the cultivation sample decrease the robustness of the microscopy method. Contrary to that, sample preparation for flow cytometry only consists of dilution steps and fluorescence staining. Data treatment is conducted with beforehand provided software files in CytoClus (CytoBuoy, Woerden, Netherlands) and Matlab scripts (MathWorks, Nattick, Massachusetts, USA), and thereby totally independent of the user.

Finally, the already addressed quantitative information gained from the flow cytometry method is evaluated as a great advantage and can be used as an at-line tool in production environments.

### Applicability of the method

The here presented flow cytometry method generates the same outputs regarding fungal morphology, as image analysis methods do so far. As described in the introduction section, morphology is a central point in filamentous biocultures. It is linked to broth viscosity which influences substrate transport and power input (Paul et al. 1994; Petersen et al. 2008). Furthermore, the productivity is linked to the morphology, which could be important for industrial production as well as process development (Papagianni 2004). On the other hand, process conditions influence the morphology (Quintanilla et al. 2015). This chain of effects is an important reason to monitor morphological parameters during process development and scale up. Apart from pharmaceutical bioprocesses, various fields of science are associated with filamentous fungi as for example environmental research on soil, water, air, and food (Prigione and Filipello Marchisio 2004).

To sum it up, the here developed flow cytometry method for morphological analysis is a fast and at-line applicable alternative to common morphology tools like image analysis. The application for a penicillin production process was presented, but applications in other bioprocesses and even other fields of investigation seem possible. The method distinguishes four morphological sub-populations and describes their morphology in more detail. Within this application, the focus was set especially on pellets.

## Electronic supplementary material

ESM 1 (PDF 296 kb).
