# Supplementary material for: Morphological analysis of the filamentous fungus Penicillium chrysogenum using flow cytometry—the fast alternative to microscopic image analysis
Source: Appl Microbiol Biotechnol. 2017 Sep 14;101(20):7675–88. doi: 10.1007/s00253-017-8475-2 (PMC5624980; doi:10.1007/s00253-017-8475-2)
Supplement: Supplementary file 1 — (PDF 296 kb). [file 253_2017_8475_MOESM1_ESM.pdf]

Applied Microbiology and Biotechnology

Morphological analysis of the filamentous fungus *Penicillium chrysogenum* using flow cytometry - the fast alternative to microscopic image analysis

Daniela Ehgartner[1,2], Christoph Herwig[1,2] and Jens Fricke*[,1,2]

*to whom the correspondence should be addressed to

[1] CD Laboratory on Mechanistic and Physiological Methods for Improved Bioprocesses, TU Wien, Vienna, Austria

[2] Research Area Biochemical Engineering, Institute for Chemical, Environmental and Biological Engineering, TU Wien, Vienna, Austria

Corresponding author:

jens.fricke@tuwien.ac.at

Tel (Office): +43 1 58801 166462

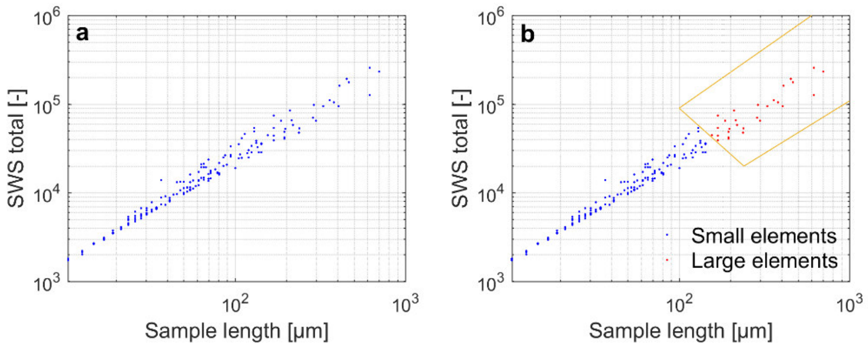

Fig. S1 Development of the gate setting. a) All hyphae and hyphal aggregates showing "natural" clustering. b) Gate setting based on "natural" clusters using the combination of visual and statistical clustering.

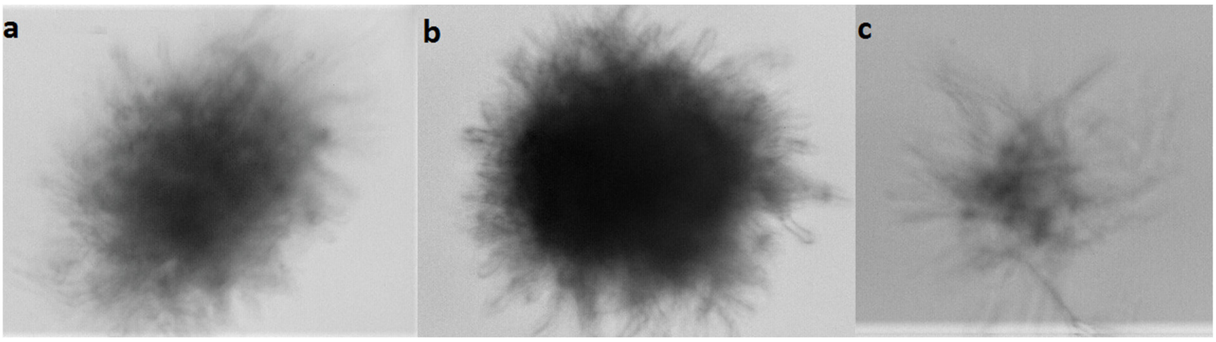

Fig. S2 Pictures of pellets taken in the flow cell of the flow cytometer. The FWS signals of these pellets are presented in Fig. 4a-c in the same order
